# Evaluation of Tooth Color Changes at Different Concentrations of Zirconia Toothpaste: A Pilot In Vitro Study

**DOI:** 10.3390/dj13100452

**Published:** 2025-10-01

**Authors:** Teuta Pustina, Besir Salihu, Miranda Stavileci, Zana Lila, Jacques Veronneau

**Affiliations:** 1 Department of Prosthodontics, Medical Faculty, University of Prishtina “Hasan Prishtina”, 10000 Pristina, Kosovo; teuta.pustina@uni-pr.edu (T.P.);; 2 Public Health Department, Faculty of Medicine, University of Prishtina “Hasan Prishtina”, 10000 Pristina, Kosovo; 3 Department of Dental Pathology and Endodontics, Medical Faculty, University of Prishtina “Hasan Prishtina”, 10000 Pristina, Kosovo; miranda.stavileci@uni-pr.edu; 4 Centres Dentaires Véronneau, Quebec, QC J0H 1K0, Canada

**Keywords:** zirconium, tooth whitening, abrasive agents, color change

## Abstract

**Background**: The demand for natural, minimally invasive teeth whitening solutions has led to interest in products using natural abrasives. Zirconium, known for its abrasive properties, has been suggested as a potential whitening agent, but its efficacy compared to traditional methods is underexplored. This study aims to address this gap by evaluating zirconium powder at various concentrations as a novel approach to tooth whitening while preserving the enamel surface. **Materials and Methods**: Forty extracted mandibular teeth (twenty anterior, twenty posterior) were allocated into four groups and brushed for 2 min with zirconia toothpaste at one of the four concentrations. Color parameters (CIE Lab*), ΔE, and WID were measured before and after brushing using a spectrophotometer; surface roughness was assessed with a profilometer. Data were analyzed with paired tests and ANOVA/Kruskal–Wallis (*p* < 0.05). **Results**: All groups showed detectable color change (ΔE > 1.2); several exceeded clinical acceptability (ΔE ≥ 2.7). WID increased in all groups, with the largest gains at 2% zirconia for posterior teeth (+31.58) and 1% or 5% for anterior teeth (+21.07, +21.19). Surface roughness decreased significantly at 0.5% (*p* ≈ 0.002) and increased at 5% (*p* ≈ 0.002); no significant change occurred at 1% and 2%. **Conclusions**: Zirconia toothpaste at 1–2% offers the best balance between whitening efficacy and enamel preservation, while 5% increases roughness and 0.5% produces smaller whitening results.

## 1. Introduction

Tooth color is a multifactorial characteristic determined primarily by the interplay between the underlying dentin, which generally has a yellowish hue, and the overlying semi-translucent enamel [1]. The dentin contributes significantly to overall tooth appearance, while enamel thickness and mineral composition modulate translucency and opacity [2,3]. Natural tooth shades vary from light yellow to grayish white [4], with aging often leading to enamel thinning, dentin exposure, and darker appearance [5]. Lifestyle habits strongly influence tooth color. Smoking introduces nicotine and tar, producing yellow-brown stains [6,7], while dietary intake of chromogenic beverages like coffee, tea, and red wine, as well as certain fruits, causes extrinsic discoloration [8]. These stains accumulate on the pellicle and enamel surface, making them amenable to mechanical or chemical removal. In contrast, intrinsic factors—such as tetracycline exposure during tooth formation, dental trauma, or fluorosis—alter the internal tooth structure and are harder to treat [9,10,11,12].

**Figure 1:**
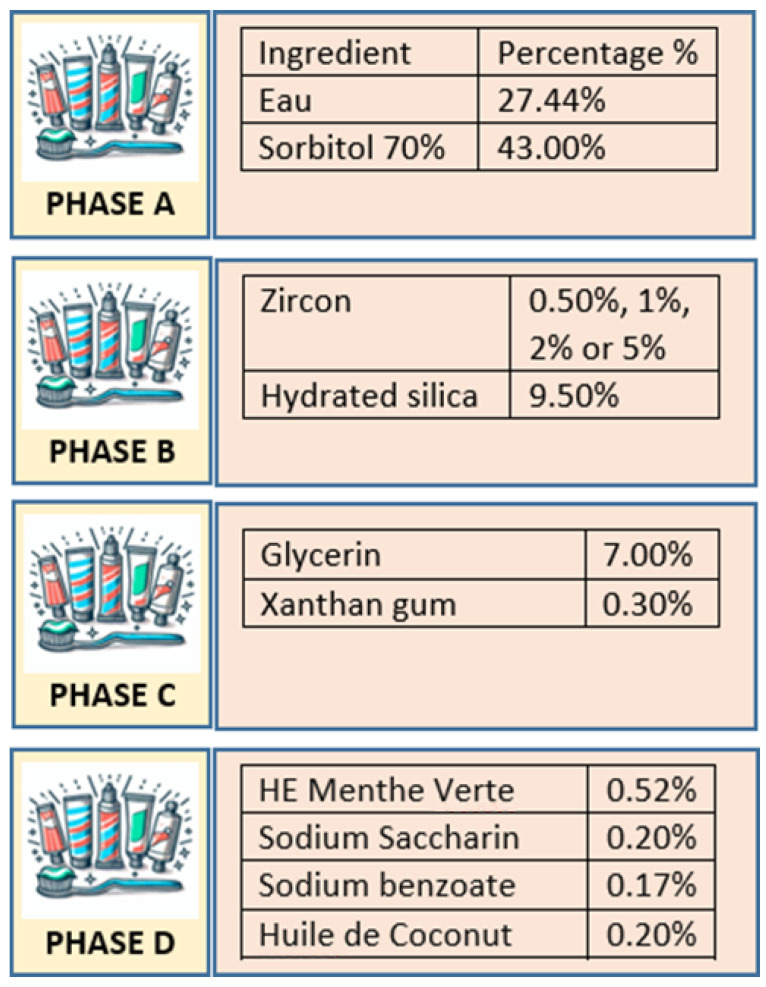
Ingredients of zirconium-containing toothpaste. Phase A incorporates solvents and humectants to provide texture, moisture, and sweetness. Phase B introduces abrasives to aid in plaque and stain removal while enhancing cleaning and whitening. Phase C focuses on maintaining moisture and ensuring the paste’s stability through thickening agents. Phase D includes flavoring, sweetening, and preservative agents to improve taste, extend shelf life, and provide additional moisturizing properties.

Patients worldwide often express dissatisfaction with their tooth shade [13,14], driving demand for whitening solutions. Conventional approaches include professional scaling, bleaching, veneers, and whitening toothpastes [15]. Whitening toothpastes primarily work by removing surface stains through abrasives such as silica [16] or by adding chemical agents like sodium tripolyphosphate to prevent and reduce staining [17]. Cosmetic pigments such as titanium dioxide or blue covarine can modify light reflection, creating the perception of whiter teeth [18]. More recently, activated charcoal has been promoted as a natural whitening abrasive, though evidence for its efficacy remains weak [18,19,20]. Despite these advances, existing abrasives may either be insufficient for stubborn extrinsic stains or cause undesirable enamel wear with prolonged use. This gap has prompted the exploration of alternative abrasives, including zirconia. Zirconia (zirconium dioxide) is a biocompatible, chemically stable, and highly wear-resistant ceramic with a fine particle size that allows effective stain removal while potentially minimizing enamel surface damage. The toothpaste tested in this study incorporates zirconia particles as its main abrasive [Figure 1], with the aim of balancing cleaning efficacy and enamel preservation.

Evaluating whitening performance requires reliable, objective measurement methods. Spectrophotometry was chosen in this study due to its high sensitivity and reproducibility compared with visual shade matching or subjective image analysis [21,22,23]. Additionally, surface roughness can influence stain adhesion and optical properties; therefore, profilometric analysis was included to assess whether zirconia abrasives altered enamel texture.

The purpose of this paper was to evaluate the effect of zirconia-based toothpaste on the color and surface roughness of natural teeth in vitro, determining the optimal zirconia concentration for effective stain removal without excessive roughening.

## 2. Materials and Methods

### 2.1. Sample Selection and Preparation

**Figure 2:**
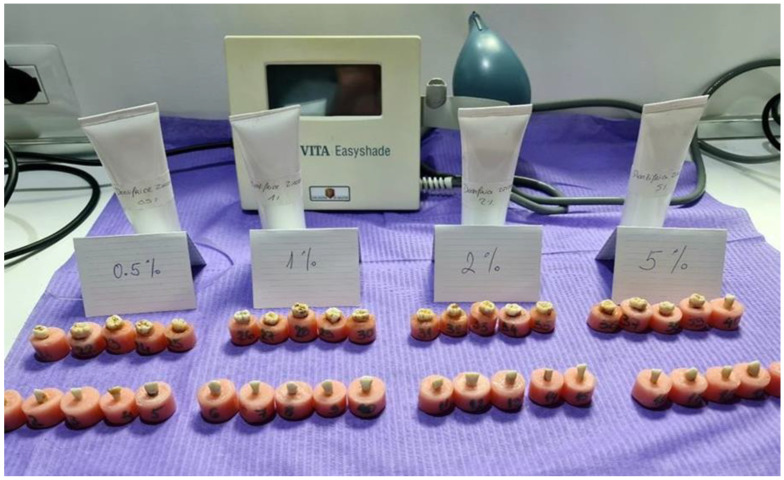
Tooth samples (5 anterior and 5 posterior), along with each toothpaste concentration in white prefabricated tubes (still in the pilot testing phase and unpatented) are shown, with the spectrophotometer visible in the background.

Forty extracted human teeth were selected for this study, consisting of twenty anterior teeth and twenty posterior teeth, all obtained from the mandibular arch. Only intact teeth without cracks, caries, restorations, or visible enamel defects were included. Teeth with intrinsic discoloration, hypoplasia, excessive wear, or structural defects were excluded. The teeth were thoroughly cleaned of debris and calculus using an ultrasonic scaler, then stored in distilled water at room temperature to prevent dehydration until further use. For stability and fixation, the roots of the natural teeth were embedded in auto polymerizing acrylic resin molds made from hollow plastic mold, up to the enamel–cementum junction, leaving the enamel of the crown fully exposed (Figure 2).

#### Toothpaste Preparation

Experimental pre-patented toothpaste formulations were produced by incorporating zirconia (zirconium oxide) particles into a base paste at four concentrations: 0.5%, 1%, 2%, and 5% by weight. The complete list of ingredients and their respective proportions is presented in Figure 1. The manufacturing process was divided into four functional phases:

• **Phase A**: Aqueous solvents and humectants (water and sorbitol) were combined to create the bulk of the base, ensuring moisture retention, texture, and a mild sweetness.
• **Phase B**: Abrasive components were introduced, consisting of zirconium oxide (in its designated concentration) and hydrated silica. These agents support stain removal and improve surface cleaning.
• **Phase C**: Glycerin and xanthan gum were incorporated to maintain moisture and provide the desired viscosity and stability.
• **Phase D**: Flavoring (peppermint essential oil), sweetener (sodium saccharin), preservative (sodium benzoate), emollient (coconut oil), and foaming agent (sodium lauryl sulfate) were added to enhance taste, extend shelf life, improve mouthfeel, and create foaming during brushing.

All phases were prepared separately under controlled laboratory conditions and combined sequentially with continuous mixing until a homogeneous paste was obtained. The final products were stored in airtight containers at room temperature until use.

### 2.2. Brushing Procedure

**Figure 3:**
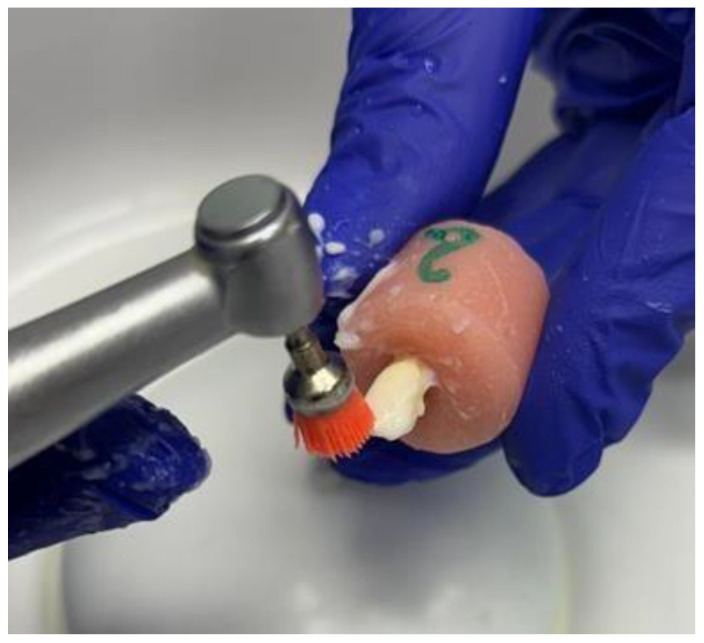
Brushing the vestibular surface of the tooth.

Each group of teeth underwent a standardized brushing protocol using a medium-bristled brush attached to a handpiece, to apply the zirconia-containing toothpaste. The teeth were randomly divided into four groups, with each group consisting of ten teeth—five anterior and five posterior. These groups were treated with one of four zirconium concentrations in the toothpaste: 0.5%, 1.0%, 2.0%, and 5.0%. Brushing was performed on the vestibular surfaces of all teeth (Figure 3), with each session lasting two minutes at a consistent speed of 20,000 rpm, ensuring uniform application across all specimens. To minimize dehydration effects, all samples were stored in distilled water until immediately before brushing, and were returned to distilled water within 1 min after the procedure. Color measurements were performed within 2 min of removal from water, in a room with controlled temperature and humidity, to reduce dehydration-induced color shift.

### 2.3. Color Measurement

**Figure 4:**
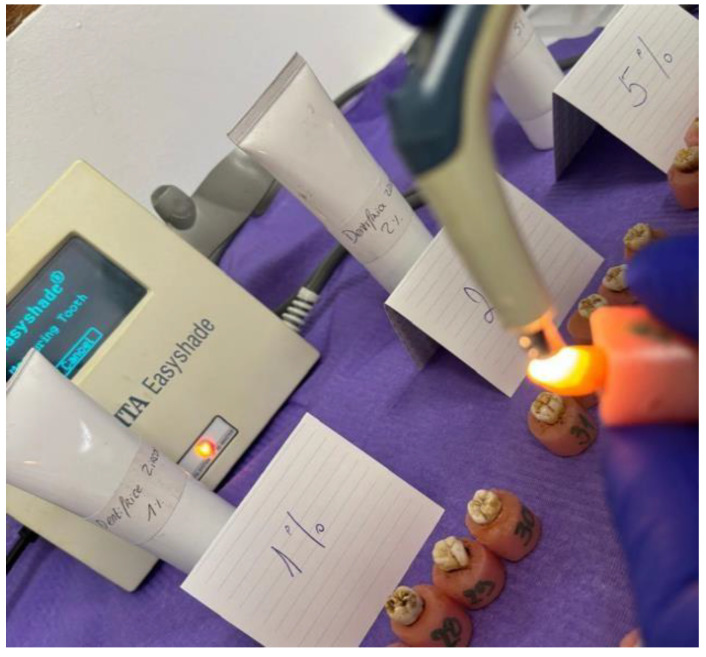
Measurement of the tooth shade using a VITA Easyshade^®^ spectrophotometer.

The tooth shade of each sample was measured before and after brushing using a VITA Easyshade^®^ spectrophotometer (VITA Zahnfabrik H Rauter GmbH and Co. KG, Bad Säckingen, Germany) (Figure 4). The device was calibrated according to the manufacturer’s instructions before each use. The “Tooth Single” mode was used, measuring the middle third of the vestibular surface. Measurements were recorded according to the CIE Lab color system*, where

L* represents lightness from 0 (black) to 100 (white);
a* represents the position on the red–green axis (positive values toward red, negative toward green);
b* represents the position on the yellow–blue axis (positive values toward yellow, negative toward blue).

### 2.4. Surface Roughness Measurements

The surface roughness (Ra) of the enamel was measured before and after brushing using a contact profilometer (Mitutoyo Surftest SJ-210, Mitutoyo Corp., Kawasaki, Japan) with an accuracy of 0.001 mm. For each specimen, three profilometric readings were taken at perpendicular orientations on the exposed enamel surface, before and after brushing with each toothpaste concentration, and the mean value was calculated. The same operator, blinded to group allocation, performed all profilometric assessments to minimize operator bias.

### 2.5. Data Analysis

**Figure 5:**
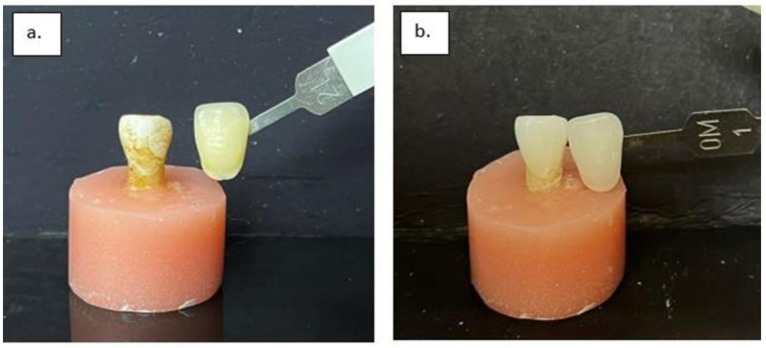
Randomly selected sample comparing the following: (**a**) the color before (2L shade) and (**b**) after cleaning with zirconia toothpaste of concentration 0.2% (0M1 shade).

The color differences (ΔE) between the initial and final measurements were calculated using the CIE Lab* color space, where ΔE represents the overall color change perceived by the human eye. The results were statistically analyzed to determine the significance of the color change in relation to the concentration of zirconia in the toothpaste (Figure 5).

The change in color (ΔE*) was calculated using the following formula:

ΔE* = [(ΔL∗)2 + (Δa∗)2 + (Δb∗)2]1/2

where

• ΔL∗, Δa∗, and Δb∗ represent the differences between the initial and final values of the L*, a*, and b* color parameters. The formula calculates the overall color change by accounting for variations in lightness (L*), the red–green axis (a*), and the yellow–blue axis (b*) following the treatment.

In addition to ΔE*ab, the Whiteness Index for Dentistry (WI_D_) was calculated for each measurement using the formula:

WI_D_ = 0.511L* − 2.324a* − 1.100b*

## 3. Statistical Analysis

The data processing was conducted using the free version of InStat. The statistical parameters calculated include the arithmetic mean, standard deviation, minimum, and maximum values. The data for the two groups (before and after application) were tested using the Paired *t*-test or Wilcoxon Matched Pairs test, depending on the data distribution. The differences between groups were compared using One-Way ANOVA and the Tukey–Kramer Multiple Comparison test when the distribution was normal, and the Kruskal–Wallis test and Dunn’s Multiple Comparison test when the distribution was not normal. The difference is considered significant if *p* < 0.05.

## 4. Results

The study included 20 anterior teeth and 20 posterior teeth, which were divided into four groups based on the concentration of the toothpaste used for brushing. Group A used 0.5% toothpaste, Group B used 1% toothpaste, Group C used 2% toothpaste, and Group D used 5% toothpaste. In each group, there were five anterior teeth or five posterior teeth. The L, a, b, C, and H values before and after brushing with the respective toothpastes are presented as means and standard deviations in Table 1 for anterior teeth and Table 2 for posterior teeth.

A significant difference in the L values for frontal teeth before and after brushing was found only at the 5% toothpaste concentration (*p* = 0.023), even though there was an increase in brightness across all groups. However, no statistically significant difference was found when comparing the L values of the anterior teeth between the different groups before and after brushing (Before *p* = 0.396; After *p* = 0.585). A significant difference in the H values before and after brushing was found at the 0.5% toothpaste concentration (*p* = 0.0003), but no significant difference was found at the other concentrations. Similarly, no statistically significant difference was found when comparing the H values of the anterior teeth between the different groups before and after brushing (Before *p* = 0.404; After *p* = 0.465). For the b and C values, a significant difference was found at three toothpaste concentrations, except for the 5% toothpaste, where there was a decrease in C from an average of 36.2 to 28.9, but the difference was not significant (*p* = 0.055), and for b from an average of 36.6 to 28.1, where the difference was also not significant (*p* = 0.063). A significant difference in the a values for anterior teeth before and after brushing was found only at the 0.5% and 1.0% toothpaste concentrations, even though there was a decrease in the mean a value across all groups.

However, no statistically significant difference was found when comparing the a, b, and C values of the anterior teeth between the different groups before and after brushing (Table 1).

For the L values of the posterior teeth before and after brushing, we found a significant difference only at the 1% and 2% toothpaste concentrations, even though there was an increase in brightness across all groups. However, when testing the L values of the posterior teeth across different groups before and after brushing, no statistically significant difference was found (Before *p* = 0.274; After *p* = 0.511). For the H values before and after brushing, a significant difference was found at the 1% and 2% toothpaste concentrations, while no significant difference was observed at the other concentrations. Similarly, when testing the H values of the posterior teeth across different groups before and after brushing, no statistically significant difference was found (Before *p* = 0.520; After *p* = 0.508).

For the b and C values, a significant difference was found only at the 0.5% toothpaste concentration. For the a values of the posterior teeth before and after brushing, a significant difference was found at the 0.5%, 1.0%, and 2% toothpaste concentrations, even though there was a decrease in the mean a value across all groups. However, when testing the a, b, and C values of the posterior teeth across different groups before and after brushing, no statistically significant difference was found (Table 2). Based on the L*, a*, and b* values, we calculated ΔE for each group, with the mean values (±SD) and range presented in Table 2. We used One-Way ANOVA to test the ΔE means across the groups and found no statistically significant difference between the anterior teeth (*p* = 0.267) and posterior teeth (*p* = 0.372). However, in Group A (toothpaste with 0.5% concentration), a statistically significant difference was found between the ΔE of the anterior and posterior teeth (*p* = 0.042). A significant difference was also found in Group B (toothpaste with 1% concentration), (*p* = 0.041). No statistically significant difference was observed between the ΔE of the anterior and posterior teeth in Group C (toothpaste with 2% concentration) (*p* = 0.170) and Group D (toothpaste with 5% concentration) (*p* = 0.757) (Table 3).

Table 4 presents the mean WI_D_ values before and after brushing for each toothpaste concentration, along with ΔWI. All groups demonstrated an increase in WI_D_ after brushing, indicating an overall improvement in tooth whiteness. The largest increases in WI_D_ were observed in the posterior teeth for the 2% zirconia toothpaste group (+31.58) and in the anterior teeth for the 1% and 5% groups (+21.07 and +21.19, respectively). According to the established perceptibility (PT = 1.2) and acceptability (AT = 2.7) thresholds for ΔE*ab (Paravina et al., 2015) [24], all mean color changes observed in the present study exceeded the PT, indicating that they were detectable to the human eye. Furthermore, several groups surpassed the AT, suggesting that these differences would be considered clinically acceptable improvements in whiteness. Similarly, evaluation of the Whiteness Index for Dentistry (WI_D_) using the thresholds reported by Pérez et al. (2019) [25] confirmed that all post-brushing increases were perceptible, with some also meeting or exceeding the acceptability limit.

Table 5 presents the mean, standard deviation, median, and range of surface roughness values (Ra, µm) before and after brushing for each group. In the posterior teeth, Group A (0.5% zirconia toothpaste) showed a significant decrease in roughness (*p* = 0.0021), while Group D (5%) exhibited a significant increase (*p* = 0.0021). Groups B (1%) and C (2%) showed no statistically significant changes. Similarly, in the anterior teeth, Group A demonstrated a significant reduction in surface roughness (*p* = 0.0020), and Group D showed a significant increase (*p* = 0.0020). No significant differences were observed for Groups B and C.

## 5. Discussion

This in vitro study evaluated the effectiveness of whitening toothpaste containing zirconia particles at four different concentrations, focusing on their impact on tooth color change and surface roughness. The goal was to determine how these abrasive ingredients contribute to whitening while also monitoring potential surface alterations, making the whitening process both effective and safe for at-home use. Aside from toothpaste formulation and active ingredient concentration, whitening outcomes can also be influenced by the teeth’s initial color. Teeth with lighter shades generally show less perceptible whitening, while darker or more yellow teeth tend to exhibit greater changes in brightness and hue [26,27]. In this study, random allocation of teeth into groups ensured even baseline color distribution across all toothpaste concentrations.

The color analysis revealed that anterior teeth showed significant improvements in brightness (L value) only at the 5% zirconia concentration and a significant shift in hue (H value) at 0.5%. Posterior teeth displayed significant L and H changes, mainly at the 1% and 2% concentrations. While reductions in the chroma (C) and yellow–blue axis (b value) were observed in several groups, statistical significance was not always achieved. Both ΔE*ab and the Whiteness Index for Dentistry (WID) confirmed perceptible whitening across all concentrations, with optimal WID gains varying between the anterior and posterior teeth—highest in the posterior teeth at 2% (+31.58) and in the anterior teeth at 5% (+21.19).

Surface roughness measurements provided further insight into these findings. All toothpaste concentrations caused some increase in Ra values after brushing, with the largest changes observed in the higher zirconia concentrations. Although these increases were statistically significant in certain groups, the final roughness values remained within clinically acceptable limits, suggesting minimal risk of adverse tactile changes or plaque retention. Interestingly, the groups with the greatest whitening improvements did not always correspond to the highest roughness increases, indicating that whitening efficacy was not solely dependent on abrasive-induced surface modification. For example, the 2% zirconia group in the posterior teeth achieved the highest ΔWI while maintaining moderate roughness changes, whereas the 5% group in the anterior teeth achieved strong whitening with a more pronounced, yet still acceptable, roughness increase.

This relationship between roughness and color change underscores the importance of balancing whitening performance with enamel preservation. Excessive abrasion can lead to surface damage, increased staining susceptibility, and tactile changes, while insufficient abrasiveness may limit whitening efficacy. The present findings suggest that zirconia-based toothpastes can achieve noticeable whitening without exceeding safe roughness thresholds, provided that the concentration and brushing technique are appropriately controlled. The whitening mechanism of zirconia particles, based on roughness values, appears to be primarily mechanical rather than chemical. Zirconia is a high-hardness ceramic (Mohs hardness ~8.5) capable of removing surface stains and pellicle more effectively than softer abrasives such as silica or calcium carbonate. Its fine particle size allows for controlled micro-polishing of enamel, producing a smoother, more light-reflective surface. This optical enhancement increases perceived brightness (L*) and whiteness index values, even when colorimetric changes in hue and chroma are moderate. Additionally, zirconia’s refractive properties may contribute to light scattering at the enamel surface, further enhancing visual whiteness without deep structural alteration.

Generally, commercial whitening toothpastes enhance tooth whiteness by gradually removing or managing extrinsic stains over time. These over-the-counter products are intended to provide patients with a slow, long-term whitening effect [28]. While it is true that most surface stains on enamel can be effectively eliminated through professional cleaning, these toothpastes offer a more accessible, at-home solution [29].

Given the growing concerns around traditional peroxide-based teeth whitening methods [28], the use of peroxide-free whitening toothpastes for at-home application has emerged as a safe and effective alternative. The toothpaste used in this study is free from hydrogen peroxide and utilizes zirconium oxide particles as the primary abrasive ingredient.

Although various whitening toothpastes with different abrasives are available on the market and have been studied by several researchers [30,31,32], direct comparison to our results is not possible, as a toothpaste with a formulation like ours has yet to be introduced. Recently, activated charcoal has gained popularity in whitening toothpastes. The way it works in teeth whitening is by adsorbing surface stains on the teeth, which helps reduce discoloration. Its porous structure traps particles and plaque, contributing to the appearance of whiter teeth.

However, activated charcoal primarily affects surface stains and may not deeply penetrate the enamel, leading to only modest improvements in tooth color [33]. Additionally, its abrasive nature can potentially wear down enamel over time if used excessively [34].

Additionally, traditional whitening toothpastes containing peroxides, baking soda, silica, as well as remineralizing pastes, have not demonstrated significant effectiveness in changing tooth color; their impact is primarily limited to surface alterations in the enamel [35]. Dursun, M. N. et al. [32] investigated the effectiveness of various whitening toothpastes with different abrasives on color improvement and concluded that all tested toothpastes demonstrated similar, clinically acceptable results in terms of enamel color change. This finding is also supported by Lima, L. C. et al., who similarly observed that, despite differences in their mechanisms of action, all toothpastes reduced tooth yellowness and achieved similar overall color [18]. After restoring front teeth with composite resin, maintaining the color and preventing discoloration over time can be challenging. Yazkan, B. et al. found whitening toothpastes effective in improving the color of stained anterior composite resin after 30 days of continuous use [36].

Several methodological limitations should be considered when interpreting the findings of this study. Firstly, brushing was performed manually using a rotating handpiece, and although efforts were made to maintain consistent brushing time and technique, variations in applied pressure and motion between samples cannot be entirely excluded. Given the relatively small sample size, such variability could have influenced both color change and surface roughness outcomes. Secondly, the possibility of enamel dehydration during the experimental procedure must be acknowledged. Brushing for two minutes, combined with the frictional heat generated by the bristles, may have led to transient surface dehydration, a factor known to increase measured brightness and whiteness beyond the true post-treatment values [37]. Although teeth were rinsed with distilled water immediately after brushing, rehydration is a slower process [38] and the short interval between brushing and color measurement may not have been sufficient to fully reverse dehydration effects. Consequently, part of the observed whitening could be attributed to temporary optical changes rather than permanent alterations to the enamel surface.

Future studies should aim to standardize brushing force through automated brushing devices, including controlled temperature monitoring during polishing, and allow adequate rehydration periods prior to post-treatment color measurements to minimize the influence of these confounding factors. While the approach was sufficient to detect perceptible whitening and surface roughness changes, additional evaluation methods such as scanning electron microscopy for surface morphology, gloss measurements, or long-term stain resistance testing could strengthen future studies and confirm the durability of zirconia’s whitening effect.

## 6. Conclusions

Zirconium-containing toothpaste shows promise as a whitening agent, with effectiveness and safety largely dependent on concentration. While higher concentrations provide stronger whitening, they may compromise enamel surface integrity. In contrast, formulations at 1–2% appear to achieve a favorable balance, offering clinically relevant whitening benefits without significantly increasing enamel roughness.

Within significant limitations of this study, the findings suggest that zirconia at moderate levels could be a viable alternative for daily whitening toothpaste. Further research is needed to confirm its long-term safety, define optimal usage protocols, and better understand its interactions with enamel over prolonged use.

## Figures and Tables

**Table 1:** Results of L, a, b, C, and H by groups among anterior teeth.

Anterior Teeth		A. TP 0.5% n = 5 (Mean ± SD)	B. TP 1% n = 5 (Mean ± SD)	C. TP 2% n = 5 (Mean ± SD)	D. TP 5% n = 5 (Mean ± SD)	*p*-Value
	Before	77.9 ± 5.8	80.0 ± 4.9	83.3 ± 3.3	79.8 ± 6.6	*p* = 0.404
H	After	81.1 ± 4.7	83.5 ± 3.9	84.9 ± 3.3	83.5 ± 2.3	*p* = 0.465
	*p*-value	*p* = 0.0003	*p* = 0.120	*p* = 0.297	*p* = 0.170	
	Before	39.7 ± 4.8	39.3 ± 3.7	37.8 ± 2.7	36.2 ± 5.8	*p* = 0.593
C	After	33.0 ± 2.4	30.4 ± 2.1	30.4 ± 0.8	28.9 ± 5.1	*p* = 0.234
	*p*-value	*p* = 0.038	*p* = 0.013	*p* = 0.002	*p* = 0.055	
	Before	78.7 ± 6.1	81.0 ± 5.0	84.3 ± 6.7	77.0 ± 9.2	*p* = 0.396
L	After	82.6 ± 4.2	86.3 ± 1.3	85.7 ± 2.7	83.8 ± 7.8	*p* = 0.585
	*p*-value	*p* = 0.070	*p* = 0.090	*p* = 0.700	*p* = 0.023	
	Before	8.7 ± 4.6	7.0 ± 3.9	4.4 ± 2.3	6.8 ± 4.7	*p* = 0.428
a	After	5.9 ± 3.5	3.5 ± 2.0	2.5 ± 1.9	3.2 ± 2.3	*p* = 0.200
	*p*-value	*p* = 0.022	*p* = 0.031	*p* = 0.067	*p* = 0.058	
	Before	39.6 ± 6.0	38.5 ± 3.2	37.5 ± 2.6	36.6 ± 5.5	*p* = 0.748
b	After	33.4 ± 3.5	29.2 ± 3.3	30.0 ± 1.9	28.1 ± 4.3	*p* = 0.138
	*p*-value	*p* = 0.006	*p* = 0.012	*p* = 0.0001	*p* = 0.063	

**Table 2:** Results of L, a, b, C, and H by groups among posterior teeth.

Posterior Teeth		A. TP 0.5% n = 5 (Mean ± SD)	B. TP 1% n = 5 (Mean ± SD)	C. TP 2% n = 5 (Mean ± SD)	D. TP 5% n = 5 (Mean ± SD)	*p*-Value
	Before	73.4 ± 6.7	67.7 ± 6.2	70.9 ± 11.1	74.7 ± 5.4	*p* = 0.520
H	After	82.9 ± 7.9	77.9 ± 6.7	83.5 ± 4.0	80.1 ± 6.6	*p* = 0.508
	*p*-value	*p* = 0.081	*p* = 0.012	*p* = 0.020	*p* = 0.325	
	Before	40.1 ± 3.6	38.2 ± 2.5	37.7 ± 1.5	43.3 ± 4.6	*p* = 0.058
C	After	33.0 ± 5.9	34.8 ± 3.3	32.6 ± 4.9	37.8 ± 10.1	*p* = 0.594
	*p*-value	*p* = 0.021	*p* = 0.149	*p* = 0.117	*p* = 0.204	
	Before	77.0 ± 6.1	67.6 ± 5.1/	73.8 ± 13.4/	77.1 ± 6.4/	*p* = 0.274
L	After	87.0 ± 3.3	84.9 ± 4.7	85.6 ± 6.3	82.7 ± 2.8	*p* = 0.511
	*p*-value	*p* = 0.007	*p* = 0.0004	*p* = 0.044	*p* = 0.118	
	Before	11.5 ± 4.7	14.4 ± 3.8	12.0 ± 6.6	11.4 ± 3.9	*p* = 0.670
a	After	4.1 ± 5.3	7.3 ± 3.9	2.9 ± 1.6	6.1 ± 3.6	*p* = 0.291
	*p*-value	*p* = 0.035	*p* = 0.009	*p* = 0.032	*p* = 0.153	
	Before	38.2 ± 3.4	35.2 ± 3.0	35.1 ± 3.2	41.7 ± 4.7	*p* = 0.036
b	After	30.4 ± 5.6	32.0 ± 5.0	31.1 ± 3.4	35.5 ± 8.8	*p* = 0.574
	*p*-value	*p* = 0.003	*p* = 0.210	*p* = 0.137	*p* = 0.074	

**Table 3:** Results of ΔE by groups among anterior and posterior teeth.

			Anterior Teeth		Posterior Teeth	
Toothpaste		n	ΔE	n	ΔE	*p*-Value
Mean ± SD			8.4 ± 3.5		15.4 ± 5.4	
A. TP 0.5%	(Rank)	5	(3.7–12.4)	5	(8.1–22.7)	*p* = 0.042
Mean ± SD			12.5 ± 4.9	5	19.5 ± 4.2	
B. TP 1%	(Rank)	5	(7.5–17.7)		(13.0–23.6)	*p* = 0.041
Mean ± SD			10.1 ± 2.1	5	16.8 ± 9.7	
C. TP 2%	(Rank)	5	(7.9–12.2)		(4.0–29.5)	*p* = 0.170
Mean ± SD			12.3 ± 3.2	5	10.7 ± 9.9	
D. TP 5%	(Rank)	5	(9.5–17.3)		(3.5–27.2)	*p* = 0.757
*p*-value			*p* = 0.267		*p* = 0.372	

**Table 4:** Whiteness Index for Dentistry (WI_D_) means before and after brushing with each toothpaste concentration.

Toothpaste Concentration	Anterior Teeth WI_before_	WI_after_	ΔWI	Posterior Teeth WI_before_	WI_after_	ΔWI
0.5%	−23.56	−8.24	+15.32	−29.40	1.49	+30.89
1%	−17.23	3.85	+21.07	−37.64	−8.78	+28.86
2%	−8.40	4.98	+13.38	−28.79	2.79	+31.58
5%	−16.72	4.47	+21.19	−32.97	−10.97	+22.00

**Table 5:** Surface roughness values (Ra, µm) before and after brushing for posterior and anterior teeth.

Group	Mean ± SD Before (µm)	Mean ± SD After (µm)	Median (Range) Before (µm)	Median (Range) After (µm)	*p*-Value	Significance
Posterior						
A (0.5%)	1.11 ± 0.96	0.43 ± 0.22	0.77 (0.46–2.85)	0.31 (0.25–0.67)	0.0021	Significant (decrease)
B (1%)	0.84 ± 0.47	0.71 ± 0.29	0.64 (0.48–1.69)	0.65 (0.45–1.20)	0.374	Not significant
C (2%)	1.14 ± 1.12	0.88 ± 0.85	0.65 (0.35–3.28)	0.50 (0.23–2.43)	0.106	Not significant
D (5%)	0.56 ± 0.43	1.04 ± 0.57	0.47 (0.18–1.27)	0.82 (0.34–1.94)	0.0021	Significant (increase)
Anterior						
A (0.5%)	1.05 ± 0.92	0.39 ± 0.18	0.74 (0.44–2.81)	0.29 (0.23–0.61)	0.0020	Significant (decrease)
B (1%)	0.80 ± 0.44	0.70 ± 0.28	0.60 (0.46–1.64)	0.64 (0.42–1.18)	0.372	Not significant
C (2%)	1.12 ± 1.10	0.85 ± 0.82	0.65 (0.33–3.21)	0.48 (0.21–2.39)	0.104	Not significant
D (5%)	0.53 ± 0.41	1.01 ± 0.55	0.45 (0.16–1.25)	0.80 (0.32–1.90)	0.0020	Significant (increase)

## Data Availability

The data presented in this study are available on request from the corresponding author.
